# Notes and News

**Published:** 1900-03-31

**Authors:** 


### Notes and News.

Hospital for Paralysed and Epileptic, Queen's Square.—Annual Meeting, at four p.m., on Tuesday, April 5th.

Samaritan Free Hospital for Women and Children.—Annual Meeting, at five p.m., on Tuesday, April 3rd.

Lord Lister has been appointed sergeant-surgeon-in-ordinary, and Mr. F. Treves surgeon-extraordinary, to the Queen.

One hundred pounds a year for five years has been promised anonymously to endow a colonial fellowship in connection with the Liverpool School of Tropical Medicine. It is to be offered to a student or graduate of a colonial university desiring to carry on bacteriological research at the Thompson-Yates laboratories.

A vice-president of the Dental Hospital of London, Leicester Square, has presented £100 as a special donation towards the amount urgently required for rebuilding the hospital, with a promise that, provided an additional £800 be collected within a reasonable time, he will give the remaining £100 to complete £1,000. The committee are earnestly appealing for contributions to enable them to avail themselves of this generous offer, and donations will be gratefully received by the secretary at the hospital, or by the bankers, Messrs. Barclay and Co. (Limited), 6, Pall Mall East, S.W.

So far the accommodation of the home military hospitals has not been overtaxed by the sick and wounded returning from the war. The military authorities do not require any of the civil hospitals to keep beds vacant for soldiers, as there will always be sufficient time to give notice before occupation is required. Many patients are now on sick furlough with their friends. Very few have availed themselves of offers of accommodation in public and private convalescent institutions. About 7,000 beds for this purpose have been offered in all parts of Great Britain, and much disappointment has been felt by some who have collected considerable funds and prepared establishments for convalescent soldiers. It is after all only natural that soldiers who have gone through danger and sickness should desire to join their friends as soon as possible. There will be ample work for the kind-hearted in supplying little comforts to Tommy in his home.

The Dublin Hospital Sunday Fund collection for the past year amounted to £4,182.

Plague is still increasing in India, and is especially prevalent in Calcutta and Bombay, where small-pox is also greatly adding to the death-rate.

In connection with Dr. Spurrier's investigation of malaria in Zanzibar a complete map of the mosquito-infested district is being made.

Dr. Hector Clare Cameron has been appointed professor of clinical surgery at Glasgow. Dr. Cameron was educated at Glasgow, became chief assistant to Lord Lister, and has since held appointments at most of the medical institutions of that city. He is president of the Faculty of Physicians and Surgeons of Glasgow.

During the absence abroad of Sir Savile Crossley, Viscount Duncannon will undertake some of the duties of the honorary secretaries of the Prince of Wales's Hospital Fund for London. All communications to the Fund should, as before, be addressed to the Honorary Secretaries, Prince of Wales's Hospital Fund, Bank of England, London, E.C.

"The City of London Directory, 1900" (Messrs. W. H. and L. Collingridge) is a large and handsome volume which may fairly be said to contain, in addition to the purely "directory" portion, all that anyone is likely to want to know about the City as it exists in this year of grace. All the information which it gives appear to have been revised and brought well up to date, and the same may be said of the splendid map which accompanies the volume.

The Scotch Hospital for South Africa will consist of corrugated iron huts lined with canvas and having wooden floors. There will be four ward buildings, each to contain 25 beds. An administrative block and small out-buildings will complete the equipment. The lighting will be by electricity, the dynamo being worked by paraffin. Röntgen ray apparatus, a soda-water making machine, a Chamberlayne Paster filter, Berkfeld field, and other filters have been provided. The staff consists of 64 persons, amongst whom are numbered five female nurses. It is expected the hospital will be erected near Bloemfontein.

At this time when England is suffering from an unusually cold spring, the detailed accounts of the heat wave which visited Buenos Ayres last month are difficult to realise. So severe was the effect on the population that at first it was reported that the victims were suffering from the plague. In one day alone 320 were suddenly seized with the alarming symptoms prevalent, which mostly proved fatal. The resources of the Assistance Publique were taxed to the utmost, and its splendid and efficient organisation received the highest praise. As horses suffered as much as human beings, the "Assistance" was soon obliged to apply to the military to supplement those they had lost, as horse ambulances were at work both day and night. All day victims fell down unconscious in the streets, and in spite of every effort to recover them most died in a few hours.

